# Carotid Endarterectomy Surgeries: A Multimodality Intraoperative Neurophysiological Monitoring Approach

**DOI:** 10.7759/cureus.26556

**Published:** 2022-07-04

**Authors:** Faisal R Jahangiri, Marie Liang, Misty Huckabey, Naomi Baloney, Sarah Sharifi

**Affiliations:** 1 Neurophysiology, Global Innervation LLC, Dallas, USA; 2 Neurology, AINeuroCare Academy, Dallas, USA; 3 Intraoperative Neuromonitoring Program, Labouré College of Healthcare, Milton, USA; 4 Neuroscience, School of Behavioral and Brain Sciences, University of Texas at Dallas, Richardson, USA

**Keywords:** tcd, electroencephalography, somatosensory evoked potentials, carotid endarterectomy, carotid, transcranial doppler ultrasound, stroke, neurophysiology, neuromonitoring, ionm

## Abstract

Patients with untreated carotid artery stenosis remain at high risk for stroke. Carotid endarterectomy (CEA) is a surgical procedure for the treatment of symptomatic and severe asymptomatic carotid stenosis. A small percentage of patients who do not have good collateral circulation are at high risk of cerebral ischemia during the cross-clamping of the carotid artery. Aspects of CEA, such as cross-clamping and routine shunting, can also carry the risk of perioperative stroke through dislodgement of emboli causing thrombosis, therefore, selective shunting is highly recommended during the CEA procedure. A multimodality approach of intraoperative neurophysiological monitoring (IONM) techniques such as somatosensory evoked potential (SSEP) and electroencephalography (EEG) can be used to monitor cerebral perfusion throughout the duration of the surgery and to predict the need for a selective shunt after cross-clamping. Additional use of transcranial Doppler (TCD) in the multimodality approach can aid in visualizing the cerebral blood flow and detecting any microemboli that may also cause a stroke. A multimodality IONM approach has been reported as more sensitive and specific for predicting and minimizing any postoperative neurological deficits.

## Introduction

Stroke remains one of the highest leading causes of disability and mortality globally, with incidence rates currently showing an upward trend as prevalence and mortality rates increased by 19.3% and 5.3%, respectively [1]. While a stroke can affect young adults, statistical research shows increased incidence in age groups above 50 years old, specifically elderly women above 75 years of age who have more than a 50% heightened likelihood of stroke compared to similarly aged men [1]. Common vascular surgical interventions such as carotid endarterectomy (CEA) and transcarotid artery revascularization (TCAR) have been devised to prevent the occurrence of stroke in patients diagnosed with significant carotid stenosis; however, these procedures also carry their own risk of intraoperative and postoperative stroke with previously reported 3.0-3.3% incidence rates of 30-day postoperative stroke or death following CEA procedures [2,3]. Most notably, intraoperative stroke risk from cerebral ischemia is heightened during the cross-clamping of the carotid artery, immediately after the opening of the cross-clamp due to the manipulation of the carotid artery, and within 24 hours postoperatively [3].

Intraoperative neurophysiological monitoring (IONM) modalities, somatosensory evoked potentials (SSEPs), and electroencephalography (EEG) can be used either independently or in conjunction with one another for proper monitoring of cerebral perfusion after cross-clamping in CEA [4]. As the practice of general shunting is discouraged due to potential embolic stroke risk, IONM is useful in determining the necessity and timing of the selective shunt by monitoring cerebral perfusion, in addition to minimizing postoperative deficit [4].

## Technical report

Patient selection

Patients with a high degree of carotid stenosis are ideal candidates for the CEA procedure to reduce future risk or recurrence of stroke. Typical guidelines for CEA recommend the procedure for symptomatic patients who have experienced one or more transient ischemic attacks (TIA) and have a severe (70% or more) degree of stenosis [5]. Symptomatic patients with a moderate (50-69%) degree of stenosis may also be ideal candidates for CEA on a case-by-case basis [6]. However, most patients are asymptomatic and often diagnosed after accidental detection of a sound in the neck heard via stethoscope and are only candidates for a CEA procedure if they show results of severe (70% or more) stenosis [7].

Anesthesia

The recommended anesthetic practice for CEA is the use of total intravenous anesthesia (TIVA). TIVA provides optimum anesthesia than low dose halogenated anesthesia for multimodality IONM due to a lesser impact on the cortical evoked potentials of SSEP and EEG, allowing for the recording of better signals [8]. TIVA protocol administers propofol and remifentanil with no muscle relaxant after intubation.

Surgical methods

**Figure 1:**
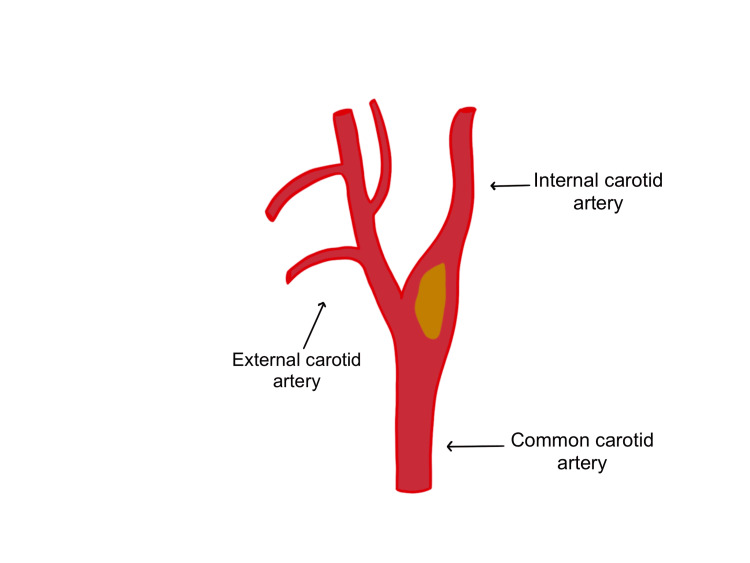
The common carotid artery splits into the external carotid artery and the internal carotid artery. The image is showing an example of a plaque which is depicted in yellow.

**Figure 2:**
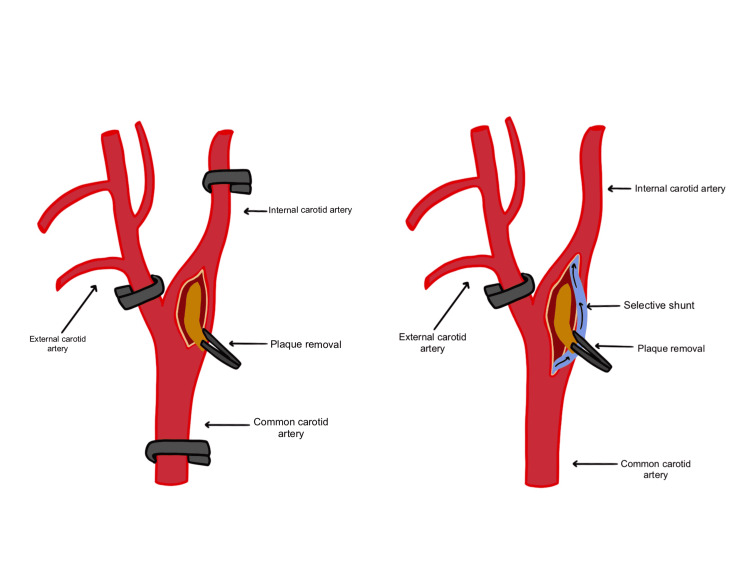
Cross-clamping of the carotid artery. Temporary clamps are placed in the order of the internal, common, and external carotid arteries. In the case of detected ischemia after clamp placement, a selective shunt (pictured in blue) should be placed in the arteries dissected.

After patient intubation, with the patient’s head tilted to the opposite side of the carotid artery surgery site, the incision is made, and temporary cross-clamping tests of the carotid arteries (internal, common, and external) are performed (Figure 1). Cross-clamping causes changes to SSEP and EEG signals and blocks all arterial branches to where there is no blood flow or reduced collateral flow to the brain (Figure 2) [8]. According to American Society of Neurophysiological Monitoring (ASNM) guidelines, approximately 69% of signal changes after cross-clamping occur within 20 seconds of placing the clamp, 80% of changes occur in less than 1 minute, and 99% of any possible changes will occur within 2 minutes after that [9].

The surgeon is notified of alarm events during continuous monitoring, and if there are any changes after a temporary clip is placed. Changes should be compared to the baseline recorded prior to clamping to determine the need for an intraluminal shunt. If any changes happen within 5 minutes, the cross-clamp should be removed immediately, and a selective intraluminal shunt should be placed within the carotid artery to improve perfusion in the circle of Willis. If no changes occur within 5 minutes, the temporary cross-clamp is removed, and this cross-clamping test should be performed at least twice before proceeding with surgery with the clamp placed. After plaque removal has taken place, the shunt and clamps are removed.

Transcarotid arterial revascularization (TCAR) is another type of carotid surgery that is gaining more popularity due to its low risk of stroke [10]. TCAR is performed with a slightly different method, where a stent is placed below the carotid so that blood flow is reversed away from the brain. Blood falls out of the artery where an incision has been made and is captured by a filter to capture the clots and other debris. This decreases the risk of stroke by sequestering plaque debris in the drainage filter. The newly filtered blood is returned to a vessel in the leg. TCAR is recommended for patients who have a higher risk of complications. Overall, TCAR differs from CEA in that it is less invasive for high-risk patients [10].

Somatosensory evoked potentials

Surface electrodes for median nerve stimulation should be placed at the wrist for upper SSEPs and for posterior tibial nerve stimulation at the medial malleolus for lower SSEPs. The stimulation pulse width should be 0.2-0.3 milliseconds (ms), the repetition rate 2.79-4.79 Hertz (Hz), and the intensity 25-30 milliamperes (mA) for the median nerve and 45-100 mA for the posterior tibial nerve.

For recording parameters, the bandpass filter is typically set to 30-500 Hz for cortical channels and 30-1500 Hz for cervical and peripheral channels. The upper extremities have a sweep that is typically set to 50 ms, and the lower extremities have a sweep of 100 ms. The display sensitivity is typically set between one and five microvolts per division. Recording electrodes are placed at Erb’s point (EP), popliteal fossa (PF), cervical vertebra five (CV5), and, according to the international 10-20 system, centro-parietal left (CP3), centro-parietal right (CP4), centro-parietal midline (CPz), and frontal midline (FPz).

**Figure 3:**
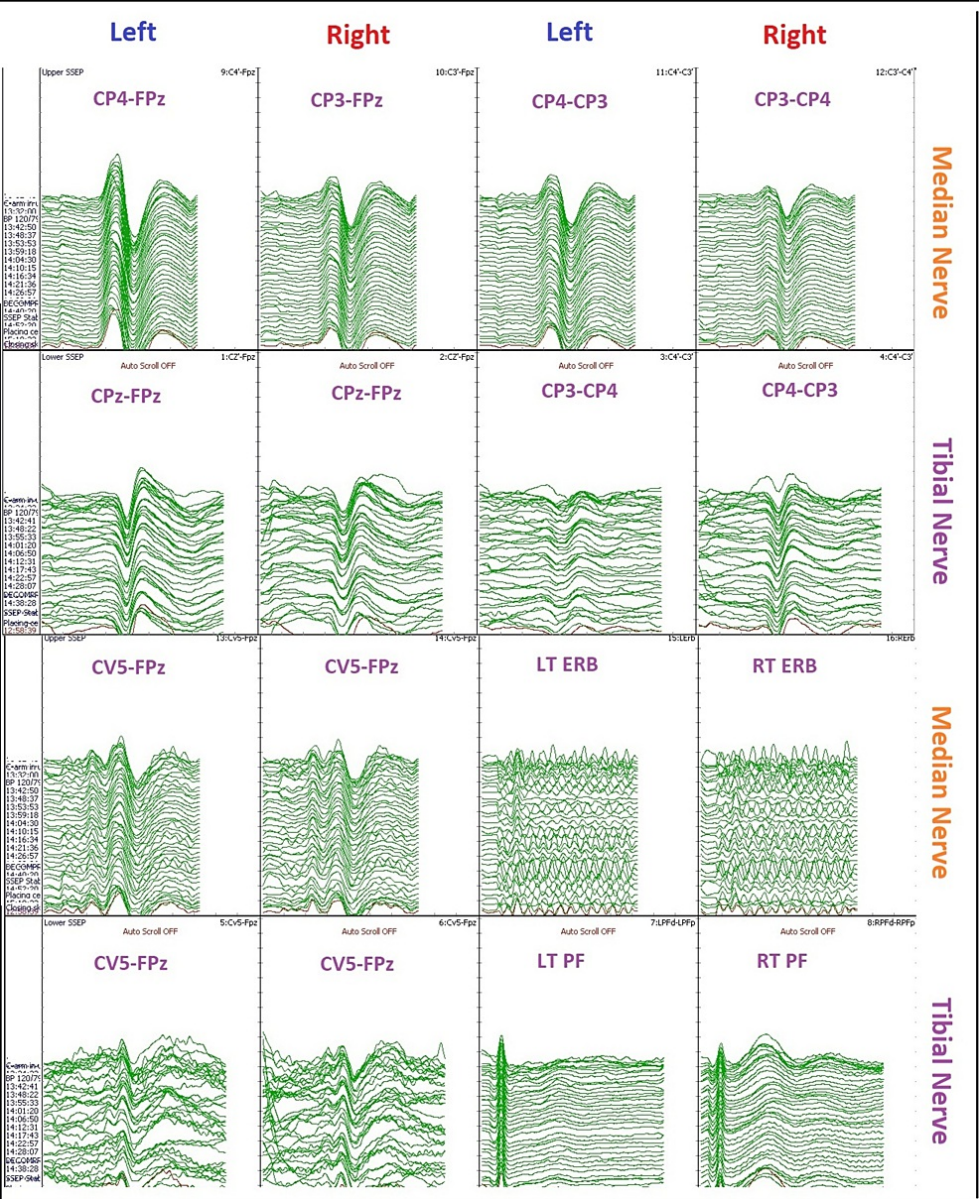
Somatosensory evoked potential (SSEP) data during carotid endarterectomy. The image is showing changes in cerebral blood flow can affect SSEP recordings. Median nerve SSEP, posterior tibial nerve SSEP, LEFT - left upper and lower extremity stimulation, RIGHT - right upper and lower extremity stimulation. CP3: centro-parietal left; CP4: centro-parietal right; CPz: centro-parietal midline; FPz: frontal midline; CV5: fifth cervical vertebra; EP: Erb's point; PF: popliteal fossa

A baseline recording should be taken prior to the surgical incision for comparison purposes and to ensure optimal stimulation intensity, and SSEPs should be performed continuously throughout the duration of the procedure. Critical periods for monitoring SSEP include before incision, before cross-clamping of the ICA, after temporary shunt placement (if shunting is needed), and for reopening of ICA [11]. The general alarm criteria of cortical SSEP amplitude (N20/P30) is a 50% or more decrease in amplitude or a 10% or more increase in latency (Figure 3) [4,8].

Electroencephalography

Recording electrodes for EEG are placed on the scalp according to the international 10-20 system, forming a minimum of eight channels when used in a multimodality approach with SSEPs and a minimum of 16 channels when used alone (Table 1) [9]. Recording parameters are a low-frequency filter of 0.3-1.0 Hz and a high-frequency filter of 70 Hz but not lower than 35 Hz without using a notch filter [9].

**Table 1:** Electroencephalography (EEG) channels. Examples of electroencephalography (EEG) channels used for carotid endarterectomy monitoring of cerebral ischemia. A minimum of eight EEG channels are required when used in conjunction with SSEP monitoring.

Left	Right
F1-C3	F2-C4
C3-O1	C4-O2
F1-T3	F2-T4
T3-O1	T4-O2

**Figure 4:**
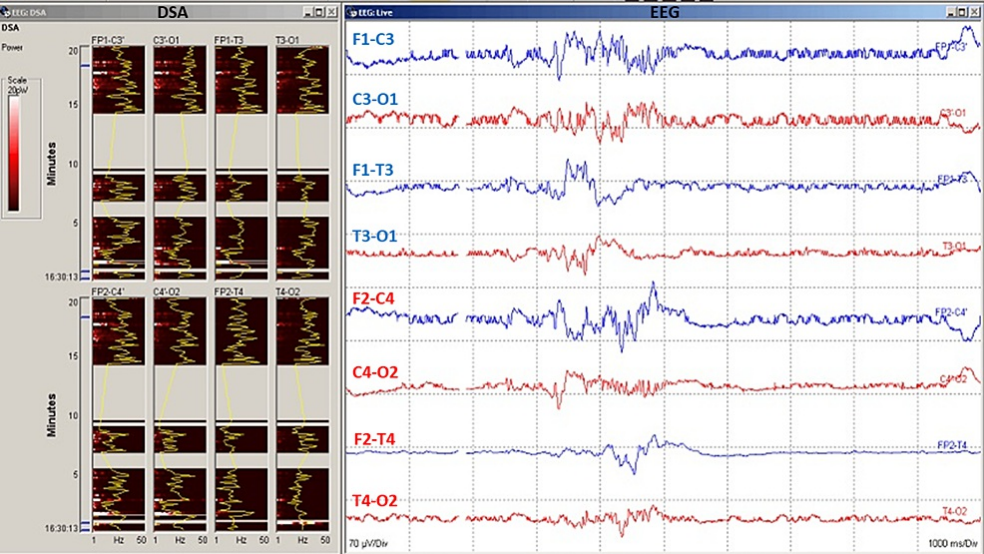
Electroencephalography (EEG) data during carotid endarterectomy (CEA) surgery. Clamp-induced changes in EEG can reflect the ischemic state of the brain. Digital spectral analysis (DSA) quantitative EEG represents EEG power vs frequency over time. EEG (raw EEG) data showing spontaneous electroencephalogram recording with burst suppression. Burst suppression should be avoided during carotid endarterectomy (CEA) for optimal monitoring of the cerebral cortex and its perfusion.

A baseline EEG recording should be taken before incision of the surgical site and continued throughout the entire duration of the CEA procedure to detect changes in cerebral perfusion. Digital EEG analysis such as compressed spectral array (CSA) or color density spectral array (CDSA) can provide additional benefits to EEG monitoring and data visualization (Figure 4). General alarm criteria for a significant change as a 50% drop of EEG amplitude (alpha) or marked ipsilateral or bilateral change in background frequency (delta) as a possible indication of cerebral ischemia [8,12]. Major EEG changes may include a more than 50% decrease in amplitude of fast-wave frequency EEG (alpha) and a more than 50% increase in slow-wave frequency (theta or delta) [4].

Transcranial Doppler

Transcranial Doppler (TCD) ultrasound provides a rapid, noninvasive, real-time measure of cerebrovascular function. TCD can be used to measure blood flow velocity in the basal arteries of the brain to assess relative changes in flow, diagnose focal vascular stenosis, or detect embolic signals within these arteries [13]. TCD can also be used to assess the physiologic health of vascular territory by measuring blood flow responses to changes in blood pressure (cerebral autoregulation), changes in end-tidal CO_2_ (cerebral vasoreactivity), or cognitive and motor activation (neurovascular coupling or functional hyperemia) [13].

**Figure 5:**
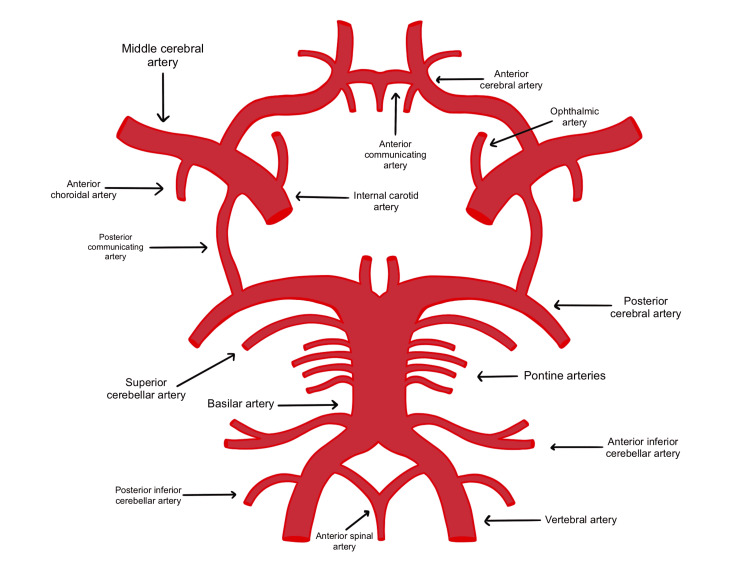
The circle of Willis forms an anastomosis that provides collateral circulation in cases of ischemic events. Patients with poor collateral circulation have a lesser degree of circulation in cases of blockage.

**Figure 6:**
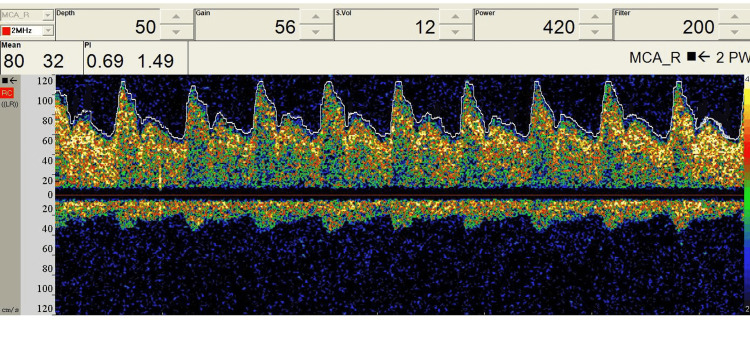
Preoperative baseline TCD data during a carotid endarterectomy surgery. TCD: transcranial Doppler

**Figure 7:**
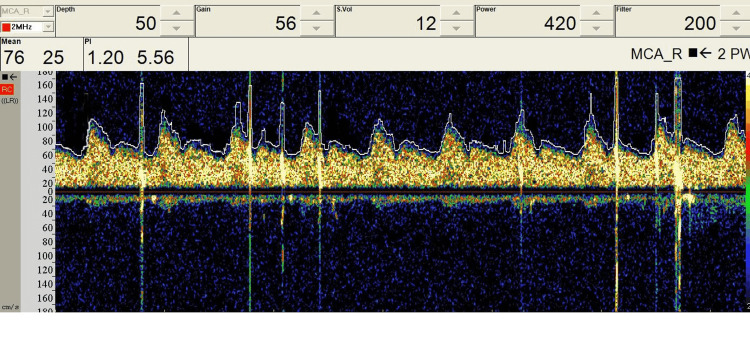
Intraoperative TCD data during a carotid endarterectomy surgery. TCD can detect the presence of microemboli that may carry stroke risk. High-intensity transient signals (HITS) are seen due to the presence of microemboli due to carotid artery manipulation. TCD: transcranial Doppler

TCD utilizes ultrasonic 2 MHz probes which can be fixed to the temporal window for middle cerebral artery monitoring to examine the circle of Willis’ perfusion (Figure 5) [13]. Optimal visualization of TCD signals requires a high-pass filter (HPF) for minimization of vessel motion artifact, and in some cases, HPF may need to be disabled in cases of low cerebral blood flow (CBF) velocity (Figure 6) [13]. High-intensity transient signals (HITS) have an amplitude above 3 dB, duration less than 300 ms, with a unidirectional acoustic sound such as a snap (Figure 7). Cross clamping may show a decrease in CBF with a more than 85% decrease denoting severe ischemia, and a 60-85% decrease denoting moderate ischemia. TCD monitoring should be used continuously throughout the procedure for measuring CBF velocity and detecting possible embolic signals [13,14].

The presence of more than one HITS per minute may be predictive of an impending stroke, and the surgeon should be alerted [13]. Previous literature has shown a significant correlation between the presence of more than 10 emboli and peri- and postoperative cerebral complications and warrants notifying the surgeon [13]. The limitations of using TCD are the need for an experienced TCD technologist and the difficulty in using it on patients without a good window.

## Discussion

The purpose of CEA is to remove plaque and improve blood supply. However, there is an increased potential for perioperative and postoperative stroke that arises from emboli or changes in cerebral perfusion during CEA surgeries. Sometimes a temporary shunt can be used to navigate insufficient cerebral perfusion during cross-clamping to remove plaque during CEA [9]. Of concern are transient ischemic attacks (TIA), which account for 87% of strokes, and can lead to a complete stroke [14]. It is imperative to have prompt recognition of stroke-related risk with a multimodality IONM approach with EEG, SSEP, and TCD. With SSEP and EEG, brain perfusion can be inferred by measuring the electrical activity of certain wavelengths relative to the patient's baseline, whereas, in TCD, sound waves are utilized to assess CBF [15]. Multimodality IONM detects cerebral changes in blood flow, which can predict the path of stroke in real-time during carotid surgery [16]. Response variability, anesthetic use, comorbidities, rate of response change, and the surgical event that occurred at the time of response change must be considered when making an intervention. Multimodality monitoring is an optimal way to accurately assess all the stroke variables during CEA.

EEG can be used to identify cortical ischemic changes that would necessitate the use of selective shunting. Selective shunting is used to avoid interruption of blood flow by bridging a clamped section of the carotid artery with a shunt and is recommended if there is poor collateral blood flow or contralateral arterial occlusion [17]. As soon as cross-clamping is enacted, EEG power may decrease on the patient’s ipsilateral side. Mild ischemia is an increase in slow frequency, moderate ischemia is a decrease in high frequency, and severe ischemia is a loss of all frequency. Burst suppression activity in EEG is caused by large doses of anesthesia and should always be avoided during the procedure. Monitoring technicians/neurophysiologists should alert the surgical team and anesthesiologist if there is an ischemic event, or the patient is in burst suppression.

Sensory evoked potentials indirectly monitor CBF for any ischemic changes. Selective shunting based on SSEP changes is typically determined by the following three factors: first, a loss of SSEP amplitude (N20/P30); second, a more than 50% reduction in SSEP amplitude after carotid cross-clamping; and third, the late and significant alterations of SSEP [9,16]. Significant changes in CBF can be identified with either SSEP or EEG [4,8]. However, a multimodality approach is best in sensitivity and specificity for monitoring blood flow during CEA for perioperative stroke risk [4,8,11].

Embolization accounts for 80% of intraoperative ischemic changes, TCD changes during CEA occur at a rate four times higher in patients who have had previous strokes compared to those without any prior strokes [18]. Many CEA-associated strokes occur after cross-clamping is released when there can be a sudden hyperperfusion mobilizing debris left behind by the removal of the plaque. This dislodgement of emboli increases the chance of cerebral ischemia and can cause unilateral head pain, delayed intracerebral hemorrhage, and may result in seizures [16]. In other cases, collateral blood flow may be very low due to stenosis or an incomplete circle of Willis. It is projected that at least 50% and up to 80% of the general population has an incomplete circle of Willis, putting many patients at an increased risk of hypoperfusion, especially during cross-clamping [9]. Transcranial Doppler (TCD) is effective at identifying debris within the artery, hyperperfusion, and hypoperfusion. The direction and speed of blood flow can be determined by trained TCD specialists. TCD is currently limited in use because there are not enough trained technologists.

Treatment for a patient with carotid artery disease depends on two factors: the severity of the blockage, and the patient's symptomatic status. The severity of blockage is best determined by a relatively quick and noninvasive carotid duplex ultrasound examination to determine the character of the plaque for future prognostic significance. Ischemic changes can be continuously monitored with transcranial Doppler ultrasound (TCD), performed at low frequency, noninvasively, through the transtemporal window [19]. High-intensity transient signals (HITS) correspond to microembolic signals and are predictors of increased stroke risk.

Multimodality cerebral monitoring using SSEP, EEG, and TCD has the highest sensitivity and specificity in detecting cerebral ischemia during and after CEA and allows for continuous monitoring of cerebral oxygen saturation [9]. Data analysis shows that when EEG and SSEP are used together, the false positive rate decreases to 3.2% in comparison to when either modality is used alone [20]. Thirty-day outcome meta-analysis found that the sensitivity of identifying a change in patients who experienced either SSEP or EEG was 1.32 times more than using EEG alone, and 1.26 times more than SSEP alone [20]. The multimodality approach is superior to the single modality approach as a tool for early intervention and in decreasing intraoperative and postoperative neurological deficits.

## Conclusions

Intraoperative modalities that enable monitoring of CBF, such as SSEP, EEG, and TCD are important in minimizing perioperative stroke risk by alerting when CBF is approaching a critical limit. Combined with the use of TCD for microemboli detection, a multimodality IONM approach utilizing both SSEP and EEG monitoring achieves a reliable means of determining the need for selective shunting, assessing the effectiveness of the shunt, and predicting complications involving sustainable blood flow that can lead to the potential risk of postoperative neurological deficit. We believe utilizing multimodality intraoperative neuromonitoring during carotid endarterectomy procedures will result in significantly decreasing perioperative stroke risk. Thus, we strongly recommend utilizing multimodality IONM as a standard-of-care practice for carotid endarterectomy procedures.
